# Supplementary material for: Long-Term Antibiotic Prophylaxis in Urology and High Incidence of Clostridioides difficile Infections in Surgical Adult Patients
Source: Microorganisms. 2020 May 28;8(6):810. doi: 10.3390/microorganisms8060810 (PMC7356804; doi:10.3390/microorganisms8060810)
Supplement: Supplementary file 1 [file microorganisms-08-00810-s001.pdf]

RELATIONSHIP OF LONG-TERM ANTIBIOTIC PROPHYLAXIS IN UROLOGY WITH *CLOSTRIDIOIDES DIFFICILE* INFECTIONS IN HOSPITALISED ADULT PATIENTS AT A MULTIPROFILE PROVINCIAL HOSPITAL IN TARNOW, POLAND.

Supplement

Depending on the type of the surgical procedure, mode of preparing the patient for surgery, and the use of implants, various regimens were employed in the antibiotic perioperative prophylaxis:

(I) **Regimen I** (antibiotic was applied only in the operating theatre)**:** clean, clean-contaminated procedures, other than the ones indicated below: cefazolin IV at a dose of 1 or 2 g (depending on the patient's weight) for up to 30 min before skin incision, the dose was repeated if the procedure was longer than 3h;

(II) **Regimen II** (antibiotics administered in the ward for up to 48 h after surgery)**:** clean procedures: vascular graft, hernia repair with mesh, mastectomy, lymphatic system, implants and spine treatments, cholecystectomy with opening of the bile duct: cefazolin IV at a dose of 1 or 2 g (depending on the patient's weight) for up to 30 min before skin incision, the dose was repeated if the procedure was longer than 3h; continuation in the ward: 1 dose 8 h after the next one and continued every 8 h;

(III) **Regimen III** (antibiotics administered in the ward for up to 72 h after surgery)**:**

A: elective procedures: large and small intestine, gastrectomy, pancreatic resection: cefazolin IV at a dose of 1 or 2 g (depending on the patient's weight) for up to 30 min before skin incision, the dose was repeated if the procedure was longer than 3h + metronidazole IV at a dose of 0.5 g min. 1 h before skin incision, the dose of metronidazole was repeated if the procedure was longer than 6 h; continuation in the ward: 1 dose 8 h after the next one and continued every 8 h;

B: elective procedures: urinary tract, TURP, pyelotitotomy: cefazolin IV at a dose of 1 or 2 g (depending on the patient's weight) for up to 30 min before skin incision, the dose was repeated if the procedure was longer than 3 h + ciprofloxacin IV at a dose of 0.4 g min. 1 h before skin incision the dose of

ciprofloxacin was repeated if the procedure was longer than 7 h; continuation in the ward: 1 dose 8 h after the next and continued every 8 h, ciprofloxacin 12 h after the last dose and continued for 12h;

C: elective procedures: implantation, revision arthroplasty: cefazolin IV at a dose of 1 or 2 g (depending on the patient's weight) for up to 30 min before skin incision, the dose of cefazolin was repeated if the procedure was longer than 3h + clindamycin IV at a dose of 0.3 g or 0.6 g (depending on the patient's weight) or vancomycin at a dose of 0.6 or 1.0 g (depending on the patient's weight) approx. 1–2 hrs before skin incision, the dose of clindamycin/vancomycin was repeated if the procedure was longer than 6 h; continuation in the ward: 1 dose respectively (cefazolin+clindamycin or cefazolin+vancomycin) 8 h after the next and continued every 8h.
